# Supplementary material for: ELF5 is a potential respiratory epithelial cell-specific risk gene for severe COVID-19
Source: Nat Commun. 2022 Aug 15;13:4484. doi: 10.1038/s41467-022-31999-6 (PMC9378714; doi:10.1038/s41467-022-31999-6)
Supplement: Supplementary file 1 — Supplementary Information [file 41467_2022_31999_MOESM1_ESM.pdf]

Supplementary Material

ELF5 is a potential respiratory epithelial cell-specific risk gene for severe COVID-19

Maik Pietzner[1,2,a], Robert Lorenz Chua[3,a], Eleanor Wheeler[2], Katharina Jechow[3], Julian D.S. Willett[4,5], Helena Radbruch[6], Saskia Trump[7], Bettina Heidecker[8], Hugo Zeberg[9,10], Frank L. Heppner[6,11,12], Roland Eils[3,13,14], Marcus A. Mall[14,15,16], J Brent Richards[4,5,17,18], Leif-Erik Sander[19], Irina Lehmann[7,14], Sören Lukassen[3], Nick Wareham[2], Christian Conrad[3,b], Claudia Langenberg[1,2,b]

Affiliations

[1]Computational Medicine, Berlin Institute of Health (BIH) at Charité – Universitätsmedizin Berlin, Germany
[2]MRC Epidemiology Unit, University of Cambridge, Cambridge, UK
[3]Center for Digital Health, Berlin Institute of Health (BIH) at Charité – Universitätsmedizin Berlin, Germany
[4]McGill Genome Centre, McGill University, Montréal, Québec Canada
[5]Lady Davis Institute, Jewish General Hospital, Montréal, Québec Canada
[6]Department of Neuropathology, Charité – Universitätsmedizin Berlin, corporate member of Freie Universität Berlin und Humboldt-Universität zu Berlin, Berlin, Germany
[7]Molecular Epidemiology Unit, Charité - Universitätsmedizin Berlin, corporate member of Freie Universität Berlin, Humboldt-Universität zu Berlin and Berlin Institute of Health (BIH), Berlin, Germany
[8]Department of Cardiology, Charité – Universitätsmedizin Berlin, corporate member of Freie Universität Berlin und Humboldt-Universität zu Berlin, Berlin, Germany
[9]Max Planck Institute for Evolutionary Anthropology, Leipzig, Germany
[10]Department of Neuroscience, Karolinska Institutet, Stockholm, Sweden
[11]Cluster of Excellence, NeuroCure, Berlin, Germany
[12]German Center for Neurodegenerative Diseases (DZNE) Berlin, Berlin, Germany
[13]Health Data Science Unit, Heidelberg University Hospital and BioQuant, Heidelberg, Germany
[14]German Center for Lung Research (DZL), associated partner site, Augustenburger Platz 1, 13353 Berlin, Germany
[15]Department of Pediatric Respiratory Medicine, Immunology and Critical Care Medicine, Charité-Universitätsmedizin Berlin, corporate member of Freie Universität Berlin and Humboldt-Universität zu Berlin, Berlin, Germany
[16]Berlin Institute of Health at Charité – Universitätsmedizin Berlin, Berlin, Germany
[17]Department of Epidemiology, Biostatistics and Occupational Health, McGill University, Montréal, Québec, Canada.
[18]Department of Twin Research, King's College London, London, United Kingdom.
[19]Department of Infectious Diseases and Respiratory Medicine, Charité - Universitätsmedizin Berlin, corporate member of Freie Universität Berlin, Humboldt-Universität zu Berlin, and Berlin Institute of Health (BIH), Berlin, Germany

[a]these authors contributed equally to this work; [b]these authors jointly supervised this work

Correspondence

Prof Claudia Langenberg (claudia.langenberg@bih-charite.de)
Prof Christian Conrad (christian.conrad@bih-charite.de)
Dr Maik Pietzner (maik.pietzner@bih-charite.de)

TABLES

Phenotype	short	cases	controls	case definition
Severe COVID-19 vs. population	A2	8,779	1,001,875	Very severeCOVID-19 cases were defined as hospitalized individuals with COVID-19 as the primary reason for hospital admission with laboratory-confirmed SARS-CoV-2 infection (nucleic acid amplification tests or serology based) and death or respiratory support (invasive ventilation, continuous positive airway pressure, bilevel positive airway pressure or continuous external negative pressure, high-flow nasal or face mask oxygen). Simple supplementary oxygen (for example, 2 L min−1 via nasal cannula) did not qualify for case status. Controls were all individuals in the participating cohorts who did not meet this case definition.
hospitalized COVID-19 vs. not hospitalized COVID-19	B1	14,480	73,191	Hospitalized COVID-19 cases were defined as individuals hospitalized with laboratory-confirmed SARS-CoV-2 infection (using the same microbiology methods as for the very severe phenotype), where hospitalization was due to COVID-19-related symptoms. Controls were all individuals with laboratory confirmed COVID-19, but did not require hospitalisation.
hospitalized COVID-19 vs. population	B2	24,274	2,061,529	Hospitalized COVID-19 cases were defined as individuals hospitalized with laboratory-confirmed SARS-CoV-2 infection (using the same microbiology methods as for the very severe phenotype), where hospitalization was due to COVID-19-related symptoms. Controls were all individuals in the participating cohorts who did not meet this case definition.
confirmed SARS-CoV-2 infection vs. population	C2	112,612	2,474,079	Susceptibility to COVID-19 cases was defined as individuals with laboratory-confirmed SARS-CoV-2 infection, health record evidence of COVID-19 (ICD coding or physician confirmation) or with self-reported infections (for example, by questionnaire). Controls were all individuals who did not meet this case definition.

Supplementary Table 1 Summary of COVID-19 endpoints from the COVID-19 Host genetics initiative - release 6

**Patient ID**	**Disease**	**Sex**	**Age**	**COVID-19 main cause of death**	**Tissue**	**SARS-CoV-2 PCR mucosa**	**SARS-CoV-2 PCR lung**
C19-1	COVID-19	Male	71-80	yes	Lung	-	pos
C19-2	COVID-19	Male	71-80	yes	Lung	-	pos
C19-3	COVID-19	Male	51-60	yes	Olf. Mucosa	pos	-
C19-4	COVID-19	Male	81-90	yes	Olf. Mucosa	neg	-
Control 1	Control	Male	31-40	no	Lung	-	-
Control 2	Control	Female	61-70	no	Lung	-	-
Control 3	Control	Female	71-80	no	Olf. Mucosa	neg	-
Control 4	Control	Female	71-80	no	Olf. Mucosa	neg	-

Supplementary Table 2 Patient characteristics

Antigen/Target	Host	Dilution	Catalog Number
KRT18	Mouse	1:100	Abcam, ab668
SFTPC	Mouse	1:100	ThermoFisher, PA5-71680
EPCAM	Mouse	1:100	ThermoFischer, MA5-12436
SCGB1A1	Mouse	1:100	Santa Cruz Biotechnology, sc-365992
ELF5	Rabbit	1:100	ThermoFisher, 720380
TMPRSS2	Mouse	1:100	Sigma, HPA035787-100UL
ACE2	Rabbit	1:100	Abcam, ab15348
AlexaFluor 488 Goat anti-Rabbit IgG (H+L)	Goat	1:500	Life Technologies, A11034
AlexaFluor 568 Goat anti-Mouse IgG (H+L)	Goat	1:500	Life Technologies, A11004

Supplementary Table 3 List of antibodies used for staining experiments

FIGURES

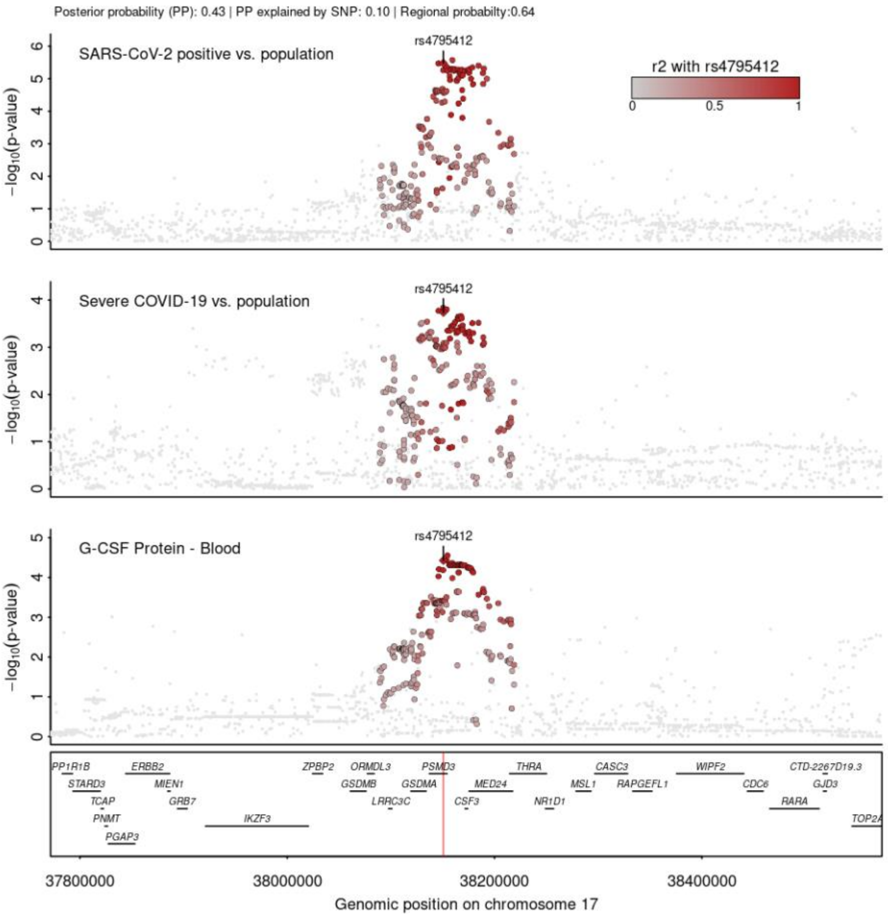

Supplementary Figure 1 Stacked regional association plots at *CSF3*. Each panel contains regional association statistics from linear regression analysis (p-values) for the trait listed in the upper left corner along genomic coordinates. Each dot represents a single nucleotide polymorphisms and colours indicate linkage disequilibrium (LD; r^2) with the most likely causative variant (rs4795412) at this locus (darker colours stronger LD). LD was calculated based on 8,350 unrelated white-British participants of the Fenland cohort.

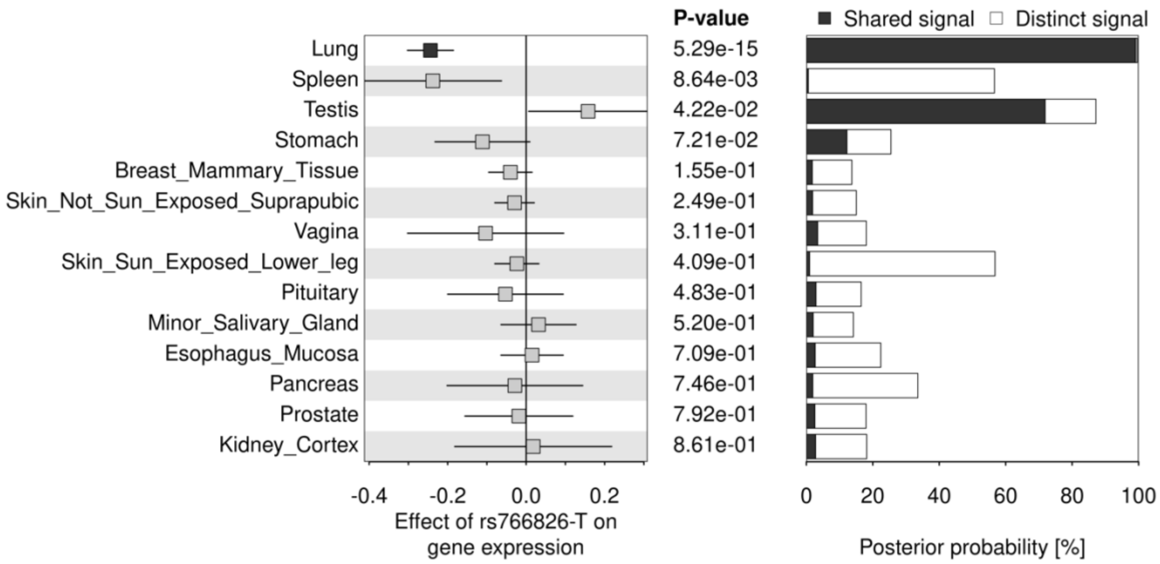

Supplementary Figure 2 Summary of cross-tissue colocalisation for ELF5. The left panel shows effect estimates (rectangles) and 95%-CIs (lines) from linear regression models for rs766826 on *ELF5* expression in all tissues with detectable levels in the GTEx v8 resource. Significant effects (p<0.001) are highlighted in black. The left-hand side shows posterior probabilities for testing for a shared genetic signal between ELF5 abundance in plasma and *ELF5* expression in each tissue using statistical colocalisation. Sample sizes from GTEx v8 were as follows: Lung (n=515), Spleen (n=227), Testis (n=322), Stomach (n=324), Breast_Mammary_Tissue (n=396), Skin_Not_Sun_Exposed_Suprapubic (n=517), Vagina (n=141), Skin_Sun_Exposed_Lower_leg (n=605), Pituitary (n=237), Minor_Salivary_Gland (n=144), Esophagus_Mucosa (n=497), Pancreas (n=305), Prostate (n=221), Kidney_Cortex (n=73)

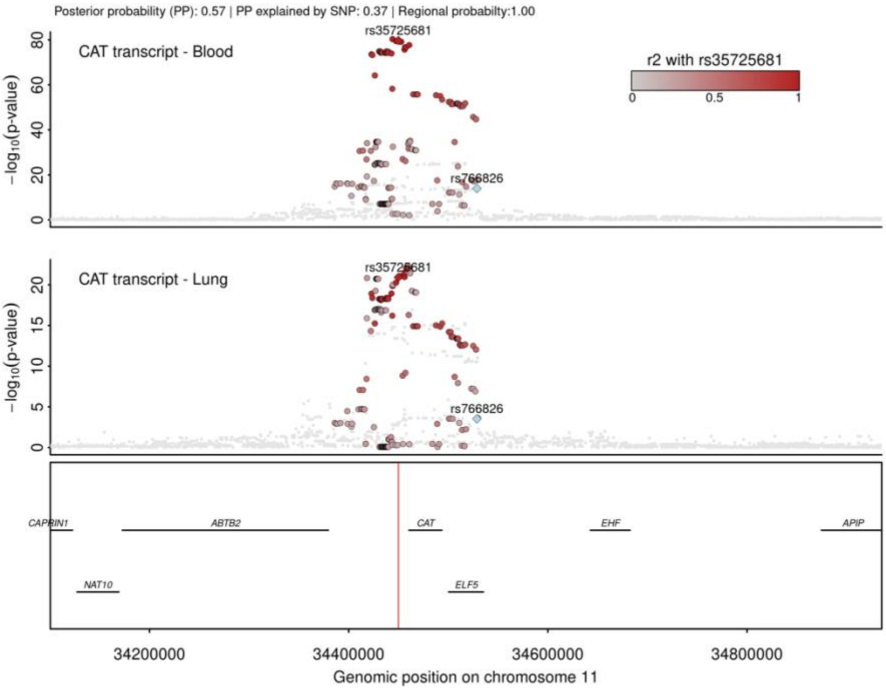

Supplementary Figure 3 Stacked regional association plots at *CAT*. Each panel contains regional association statistics from linear regression analysis (p-values) for the trait listed in the upper left corner along genomic coordinates. Each dot represents a single nucleotide polymorphisms and colours indicate linkage disequilibrium (LD; r^2) with the most likely causative variant (rs35725681) at this locus (darker colours stronger LD). Summary statistics have been obtained from the GTEx v8 consortium[1]. The most likely causal variant for COVID-19-related outcomes, rs766826, is highlighted in blue. LD was calculated based on 8,350 unrelated white-British participants of the Fenland cohort.

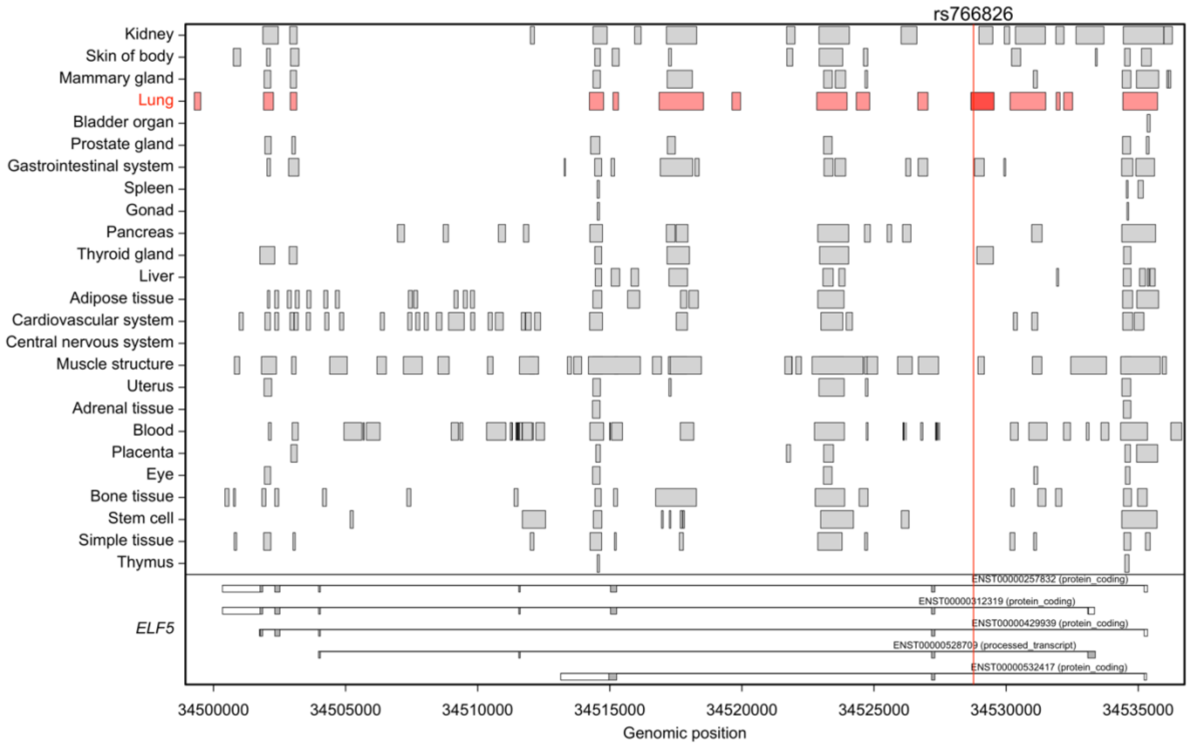

Supplementary Figure 4 Open chromatin regions at *ELF5* across various tissues. Each colour bar indicates open chromatin regions identified using ATAC-seq experiments and tissues are ordered by *ELF5* expression according to GTEx v8. The relevant lung-specific region is highlighted in red. Data was obtained from https://t2d.hugeamp.org/variant.html?variant=rs766826 and open chromatin regions in lung are based on experiments in alveolar type 2 cells (https://cmdga.org/annotations/DSR063NOE/). Position of all available *ELF5* transcripts from ENSEMBL are depicted underneath. The position of rs766826 is highlighted by a red bar.

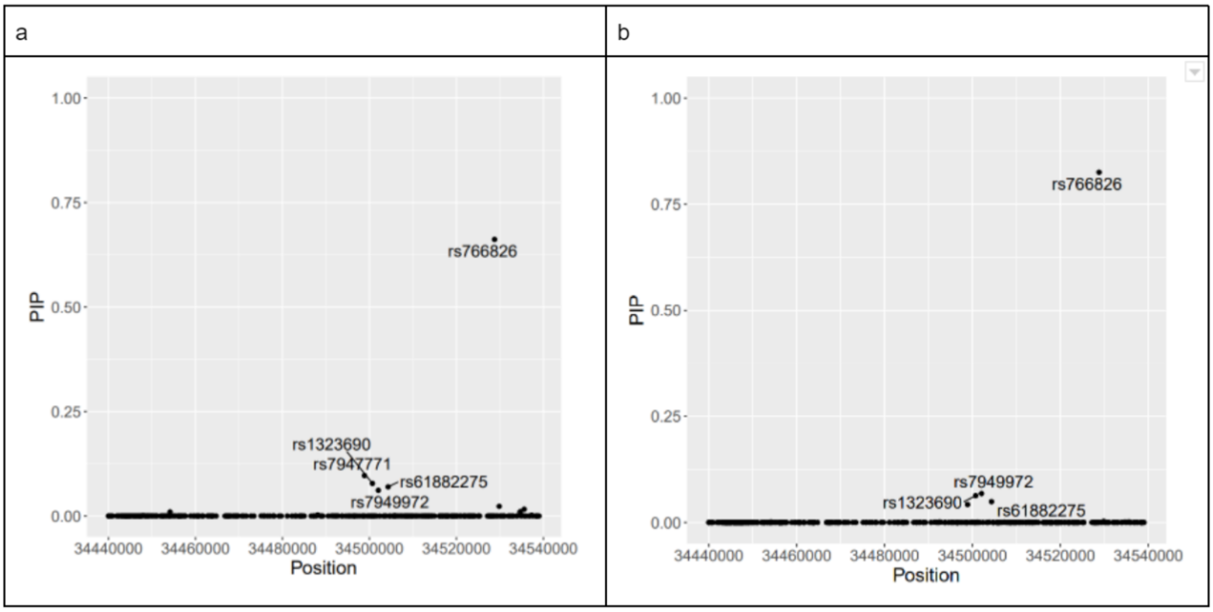

Supplemental Figure 5 Results of multi-ethnic fine-mapping at the *ELF5* locus. Posterior inclusion probabilities from fine-mapping based on European (a) and African (b) ancestry results are shown for each biallelic variants at the *ELF5* locus (chr11:34440000-34540000). Variants included in the 95%-credible set are annotated.

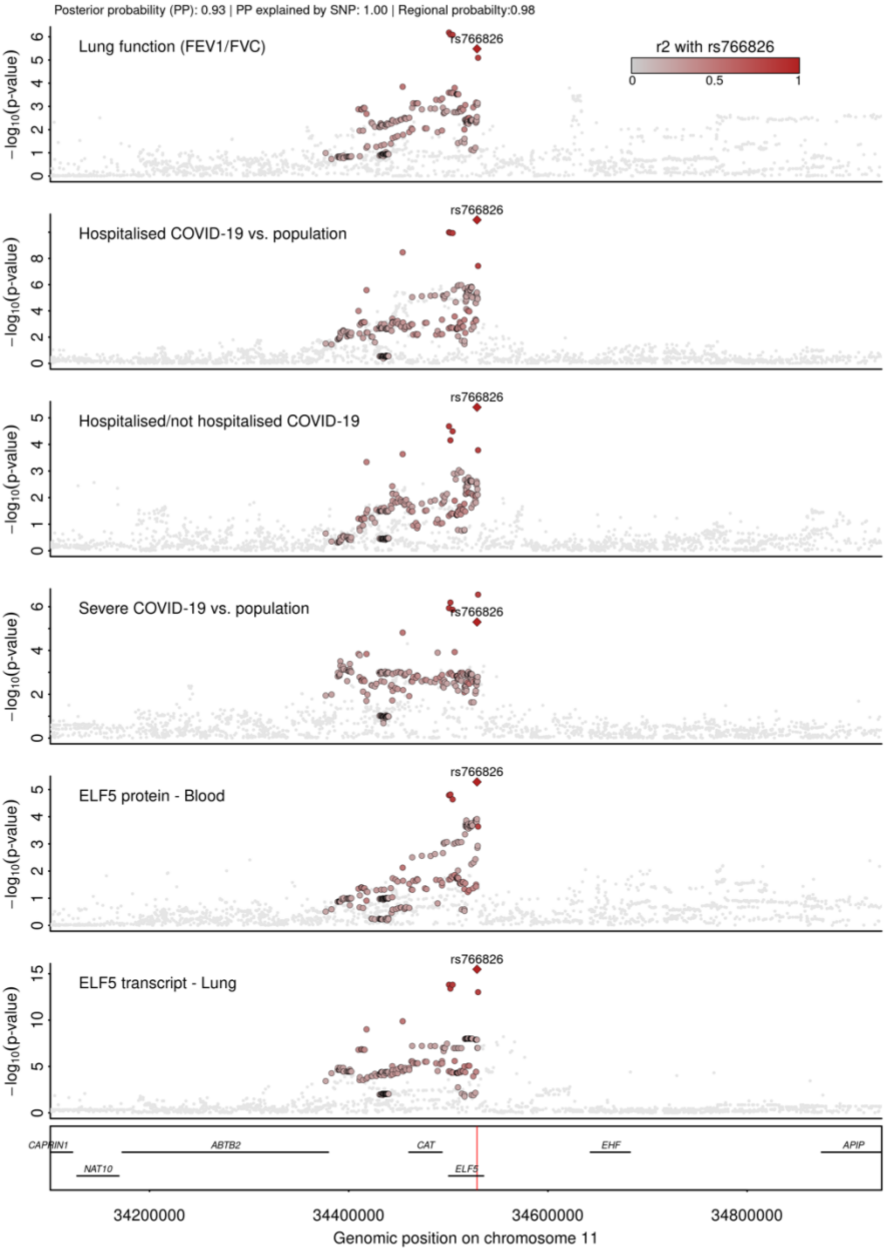

Supplementary Figure 6 Stacked regional association plots at *ELF5*. Each panel contains regional association statistics (p-values) for the trait listed in the upper left corner along genomic coordinates. Each dot represents a single nucleotide polymorphisms and colours indicate linkage disequilibrium (LD; r^2) with the most likely causative variant (rs766826) at this locus (darker colours stronger LD). This figure is similar to Figure 2 in the main text, but now also including summary statistics for the FEV1/FCV ratio as a measure of lung function taken from Shrine *et al.*[2]. LD was calculated based on 8,350 unrelated white-British participants of the Fenland cohort.

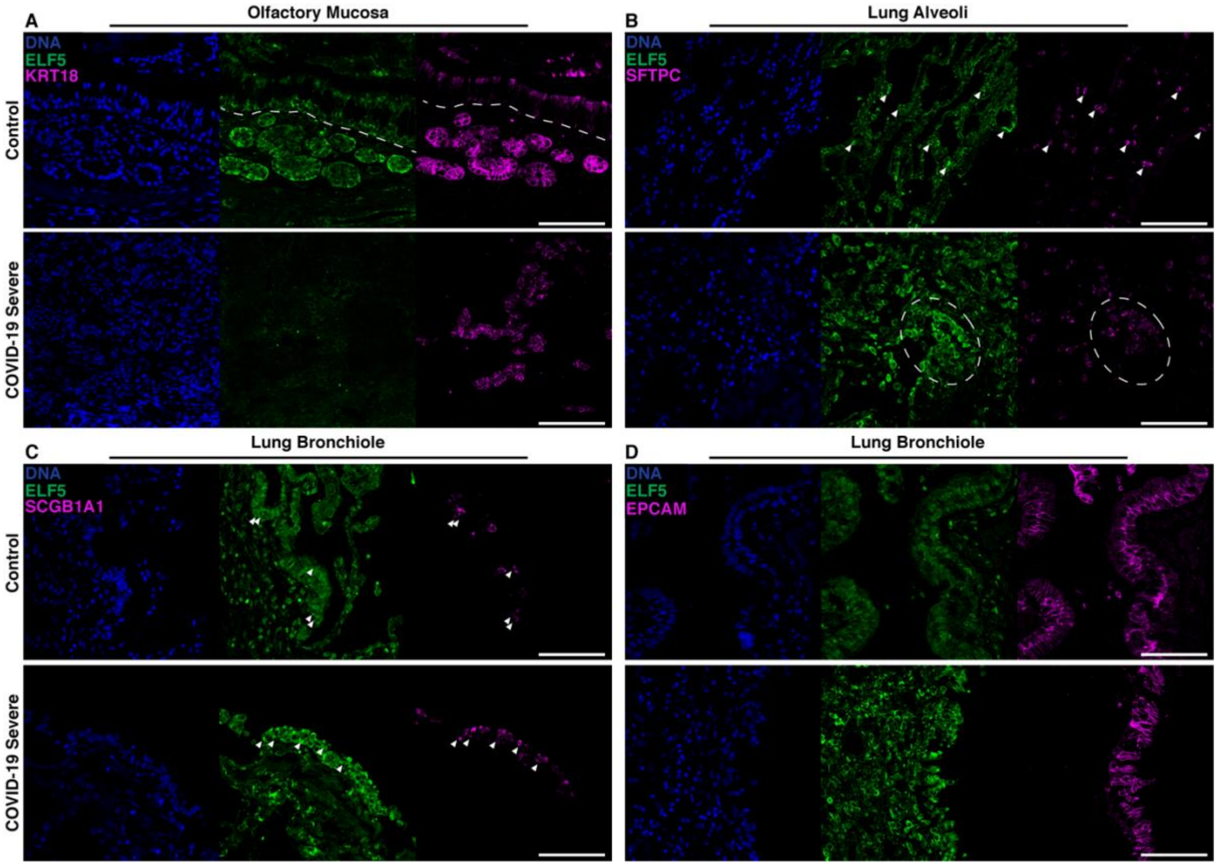

Supplementary Figure 7 ELF5 expression by epithelial cells of the olfactory mucosa and lung. Immunofluorescent staining of ELF5 in separate channels in control and COVID-19 patients in the **A** olfactory mucosa, **B** lung alveoli, and **C-D** lung bronchiole. **A** Dashed lines separate the olfactory epithelium and the lamina propria. **B** Arrowheads highlight AT2 cells expressing ELF5; dashed outline highlights clusters of AT2 cells expressing ELF5. **C** left: Arrowheads highlight secretory cells expressing ELF5; right: arrowheads highlight airway epithelial cells expressing ELF5. Marker genes for sustentacular and Bowman gland cells (**A**, KRT18), alveolar type II cells (**B**, SFTPC), secretory cells (**C**, SCGB1A1), and epithelial cells (**D**, EPCAM) are shown in purple. Validation staining for each tissue: control (n = 2); COVID-19 (n = 2). Scale bar = 100µm.

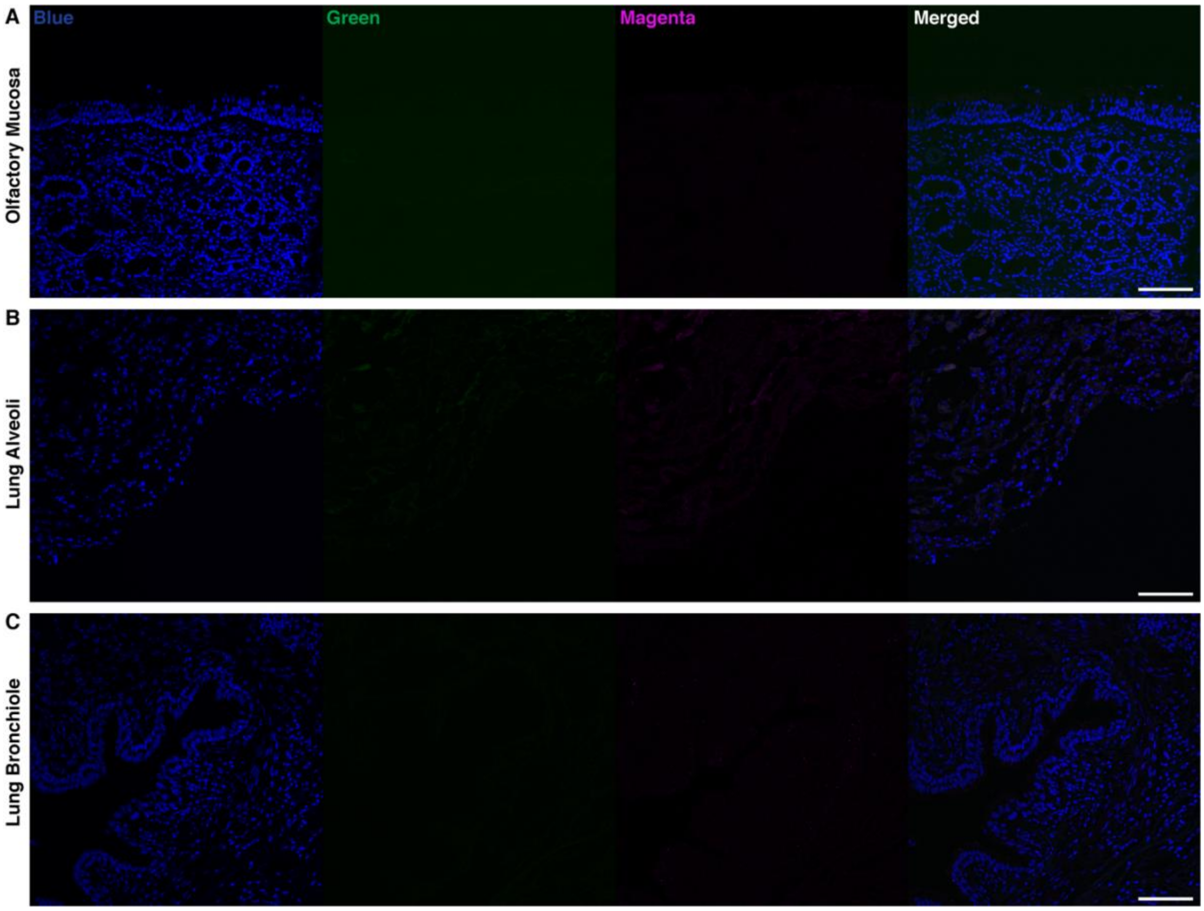

Supplementary Figure 8. No primary antibody controls of the olfactory mucosa and lung. Representative images of negative controls for the **A** olfactory mucosa, **B** lung alveoli, and **C** lung bronchiole under high laser power. Validation staining for each tissue: control (n = 2). Scale bar = 100µm.

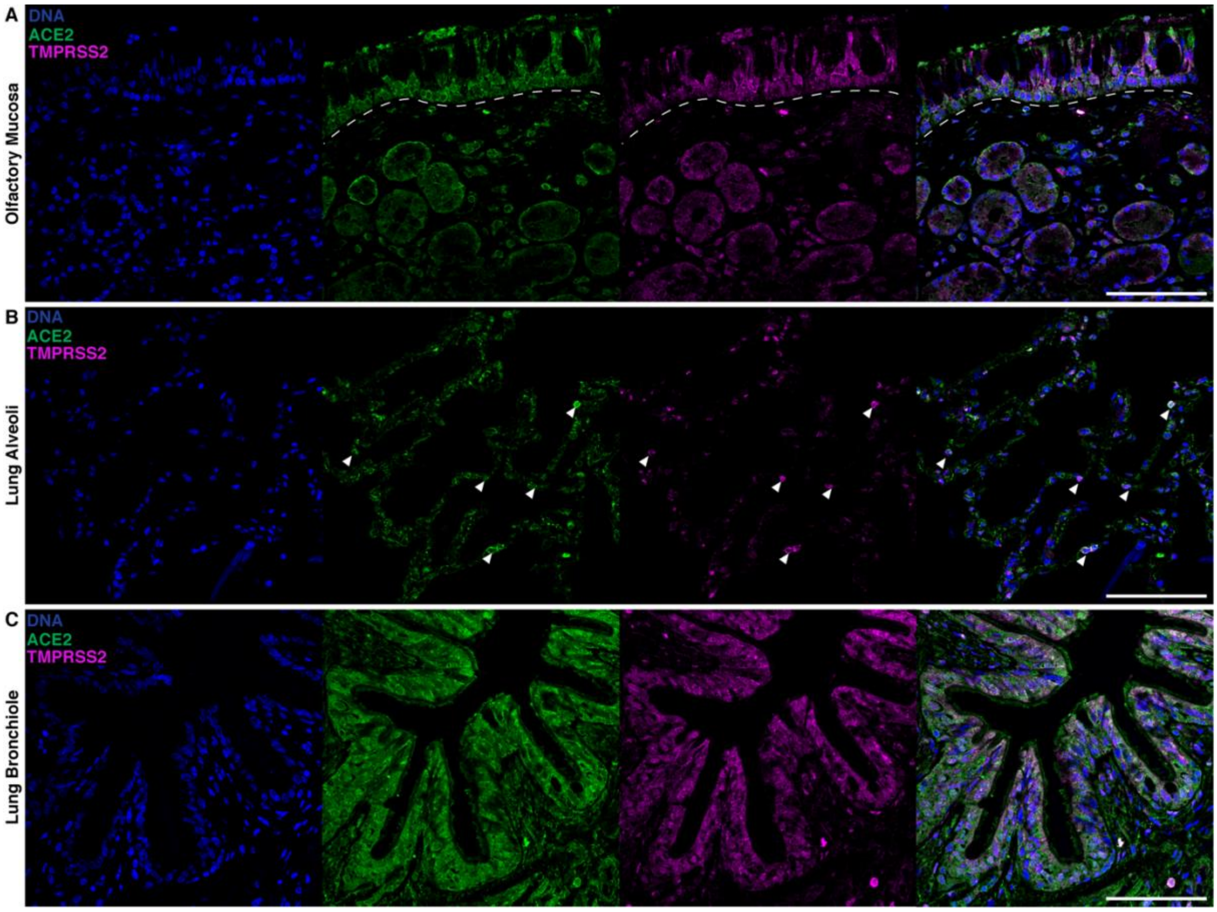

Supplementary Figure 9. ACE2 and TMPRSS2 are expressed by different epithelial cells of the olfactory mucosa and the lung. A Sustentacular cells, horizontal basal cells, and Bowman gland cells of the olfactory mucosa co-express ACE2 and TMPRSS2. **B** Arrows highlight punctuated expression of TMPRSS2 reflecting the distribution of AT2 cells together with ACE2. **C** Airway epithelial cells of the bronchioles expressing ACE2 and TMPRSS2. Validation staining for each tissue: control (n = 2). Scale bar = 100µm.

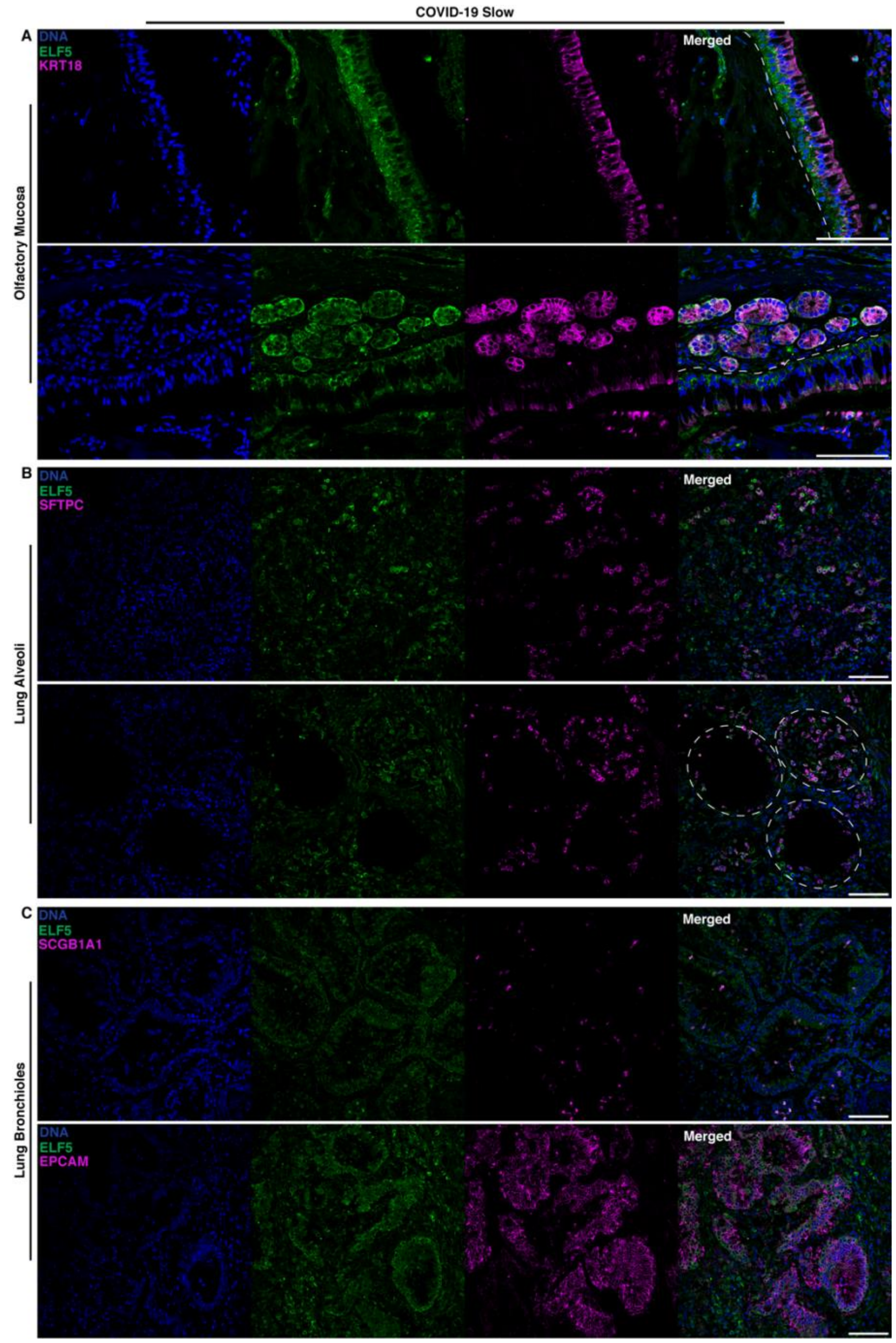

Supplementary Figure 10. ELF5 expression by epithelial cells of the olfactory mucosa and lung in COVID-19 cases with longer disease duration. Immunofluorescent staining of ELF5 in separate channels of COVID-19 patients that died after hospitalization after 14 days. ELF5 expression in the **A** olfactory mucosa, **B** lung alveoli, and **C** lung bronchiole. **A** Dashed lines separate the olfactory epithelium and the lamina propria. **B** dashed outline highlights clusters of AT2 cells expressing ELF5. Marker genes for sustentacular and Bowman gland cells (**A**, KRT18), alveolar type II cells (**B**, SFTPC), secretory cells (**C**, SCGB1A1), and epithelial cells (**C**, EPCAM) are shown in purple. Validation staining for each tissue: control (n = 2); COVID-19 (n = 2). Scale bar = 100µm.

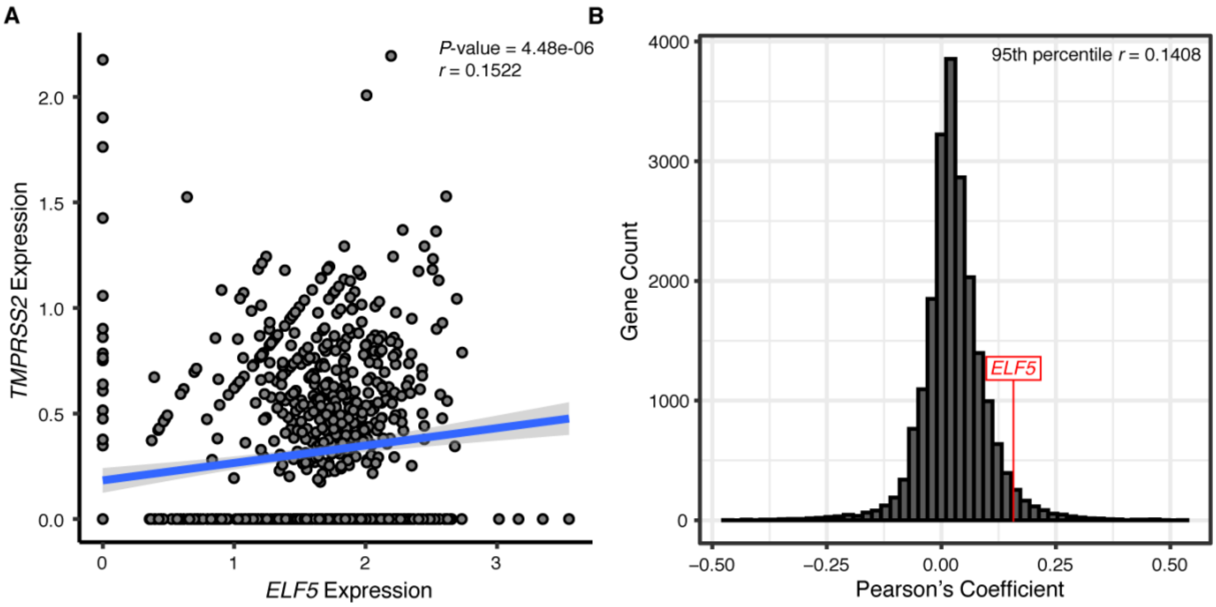

Supplementary Figure 11. Co-expression of ELF5 and TMPRSS2. A Scatterplot opposing normalised expression levels of ELF5 and TMPRSS2 in sustentacular cells of the olfactory mucosa. The blue line indicates a linear regression fit (error band indicating a 95% confidence level interval) and correlation coefficient and p-value are given in the legend. B Distribution of correlation coefficients of pairwise gene expression across all genes detected in sustentacular cells. The red line indicates were the ELF5 – TMPRSS2 correlations is placed.

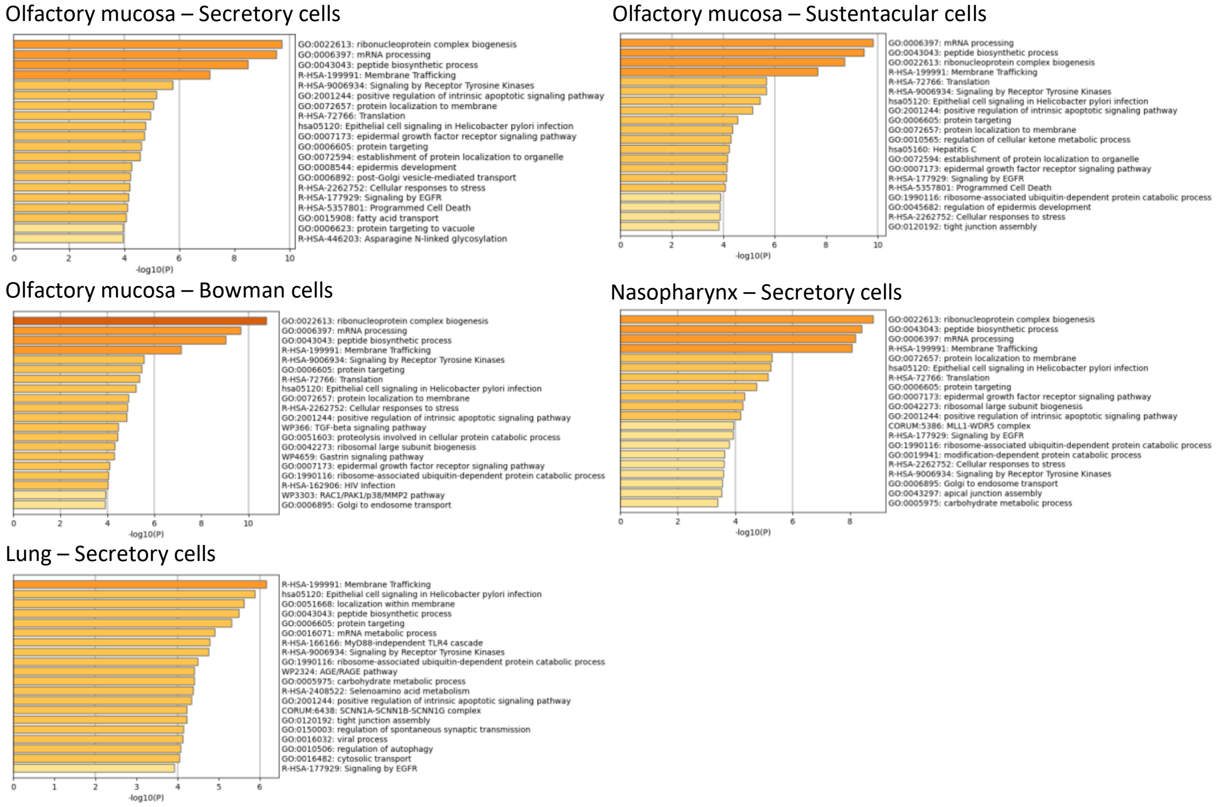

Supplementary Figure 12. Results from cell-type specific pathway enrichment analysis for predicted ELF5 targets.

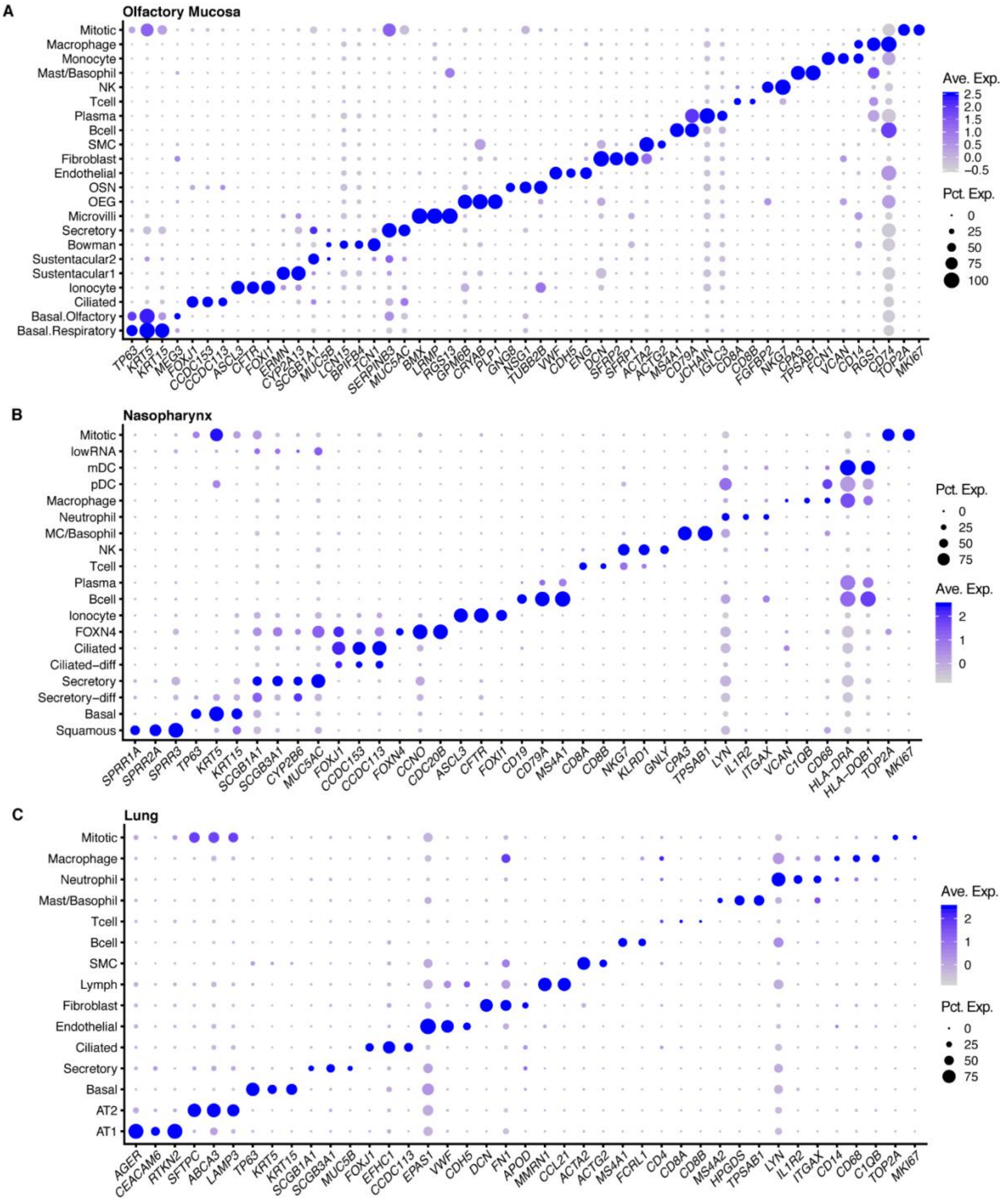

Supplemental Figure 13. Cell-specific expression of marker genes used for single cell annotation across the three tissue data sets used: **A** olfactory mucosa, **B** nasopharynx, and **C** lung. Higher expression levels are indicated by darker shades and the size of the circle reflects the percentage of cells expressing the corresponding marker gene.
