# Supplementary material for: ELF5 is a potential respiratory epithelial cell-specific risk gene for severe COVID-19
Source: Nat Commun. 2022 Aug 15;13:4484. doi: 10.1038/s41467-022-31999-6 (PMC9378714; doi:10.1038/s41467-022-31999-6)
Supplement: Supplementary file 3 — Description of Additional Supplementary Files [file 41467_2022_31999_MOESM3_ESM.pdf]

Description of Additional Supplementary Files

File Name: Supplementary Data 1
Description: Results from proteome-wide colocalisation analysis at protein-encoding loci for severe COVID-19 (A2).

File Name: Supplementary Data 2
Description: Results from proteome-wide colocalisation analysis at protein-encoding loci for hospitalised COVID-19 (B1, vs non-hospitalised COVID-19).

File Name: Supplementary Data 3
Description: Results from proteome-wide colocalisation analysis at protein-encoding loci for hospitalised COVID-19 (B2, vs population).

File Name: Supplementary Data 4
Description: Results from proteome-wide colocalisation analysis at protein-encoding loci for SARS-CoV-2 positive(C2).

File Name: Supplementary Data 5
Description: Summary from proteome-wide colocalisation screens and Mendelian randomisation analysis.

File Name: Supplementary Data 6
Description: Results from sensitivity analysis for statistical colocalisation of candidate proteins using a grid of prior settings.

File Name: Supplementary Data 7
Description: Collation of genes possible regulated or co-expressed with ELF5.

File Name: Supplementary Data 8
Description: Summary of phenome-wide colocalisation screen at CSF3
