# Supplementary material for: ELF5 is a potential respiratory epithelial cell-specific risk gene for severe COVID-19
Source: Nat Commun. 2022 Aug 15;13:4484. doi: 10.1038/s41467-022-31999-6 (PMC9378714; doi:10.1038/s41467-022-31999-6)
Supplement: Supplementary file 12 — Reporting Summary [file 41467_2022_31999_MOESM12_ESM.pdf]

nature portfolio

Corresponding author(s): Claudia Langenberg, Christian Conrad, and Maik Pietzner

Last updated by author(s): Jul 1, 2022

Reporting Summary

Nature Portfolio wishes to improve the reproducibility of the work that we publish. This form provides structure for consistency and transparency in reporting. For further information on Nature Portfolio policies, see our Editorial Policies and the Editorial Policy Checklist.

Statistics

For all statistical analyses, confirm that the following items are present in the figure legend, table legend, main text, or Methods section.

n/a	Confirmed	
☐	☒	The exact sample size (*n*) for each experimental group/condition, given as a discrete number and unit of measurement
☐	☒	A statement on whether measurements were taken from distinct samples or whether the same sample was measured repeatedly
☐	☒	The statistical test(s) used AND whether they are one- or two-sided<br>*Only common tests should be described solely by name; describe more complex techniques in the Methods section.*
☒	☐	A description of all covariates tested
☐	☒	A description of any assumptions or corrections, such as tests of normality and adjustment for multiple comparisons
☐	☒	A full description of the statistical parameters including central tendency (e.g. means) or other basic estimates (e.g. regression coefficient) AND variation (e.g. standard deviation) or associated estimates of uncertainty (e.g. confidence intervals)
☐	☒	For null hypothesis testing, the test statistic (e.g. F, t, r) with confidence intervals, effect sizes, degrees of freedom and P value noted<br>*Give P values as exact values whenever suitable.*
☐	☒	For Bayesian analysis, information on the choice of priors and Markov chain Monte Carlo settings
☒	☐	For hierarchical and complex designs, identification of the appropriate level for tests and full reporting of outcomes
☐	☒	Estimates of effect sizes (e.g. Cohen's d, Pearson's r), indicating how they were calculated

Our web collection on statistics for biologists contains articles on many of the points above.

Software and code

Policy information about availability of computer code

Data collection	No software has been used for data acquisition.
Data analysis	R v3.6.0 (packages: coloc v4.0.4, ieugwasr v0.1.5, hyprcoloc v1.0, LDLinkR v1.0), SoupX v1.4.5, Seurat v3.1.4, GSEA v4.1.0, Bcftools v1.9; Plink v2.00a22.3LM; Tomahawk v0.7.1; FIJI v.1.0<br>Associated code and scripts for the analysis will be made available on GitHub upon publication (https://github.com/pietznerm/elf5_covid19).

For manuscripts utilizing custom algorithms or software that are central to the research but not yet described in published literature, software must be made available to editors and reviewers. We strongly encourage code deposition in a community repository (e.g. GitHub). See the Nature Portfolio guidelines for submitting code & software for further information.

Data

Policy information about availability of data

All manuscripts must include a data availability statement. This statement should provide the following information, where applicable:

- Accession codes, unique identifiers, or web links for publicly available datasets
- A description of any restrictions on data availability
- For clinical datasets or third party data, please ensure that the statement adheres to our policy

Summary statistics for protein levels are available from https://omicscience.org/apps/pgwas/. Summary statistics for COVID-19 are available from https://www.covid19hg.org/results/r6/. scRNAseq data sets are available under the accession IDs: olfactory mucosa (GSE139522), nasopharynx (EGAS00001005461), and lungs (EGAS00001004689, EGAS00001004419, SAMEA6848756, SAMEA6848761, SAMEA6848765, SAMEA6848766). Publicly available GWAS summary statistics were obtained from the IEU Open GWAS project (https://gwas.mrcieu.ac.uk/).

Field-specific reporting

Please select the one below that is the best fit for your research. If you are not sure, read the appropriate sections before making your selection.

☒ Life sciences ☐ Behavioural & social sciences ☐ Ecological, evolutionary & environmental sciences

For a reference copy of the document with all sections, see nature.com/documents/nr-reporting-summary-flat.pdf

Life sciences study design

All studies must disclose on these points even when the disclosure is negative.

Sample size	No sample size calculation was performed. We choose for each analysis the largest available data set: Genome-wide summary statistics for plasma protein levels have been obtained from Pietzner et al. Science 2021 and Pietzner et al. 2021 Nature Communications. Single-cell/nuclei RNA sequencing data has been obtained from Lukassen et al. EMBO 2020, Loske et al. Nature Biotechnology 2021, Gassen et al. Nature Communication 2021, and Durante et al. Nature Neuroscience 2020.
Data exclusions	No data were excluded from the analyses.
Replication	The COVID-19 risk increasing variant mapping to ELF5 has been replicated in by two independent groups: Kousathanas, A. et al. Whole genome sequencing identifies multiple loci for critical illness caused by COVID-19. medRxiv 2021.09.02.21262965 (2021) and Pereira, A. C. et al. Genetic risk factors and Covid-19 severity in Brazil: results from BRACOVID Study. medRxiv 2021.10.06.21264631 (2021) doi:10.1101/2021.10.06.21264631.
Randomization	No experiments that require randomization were performed.
Blinding	No experiments that require blinding were performed.

Reporting for specific materials, systems and methods

We require information from authors about some types of materials, experimental systems and methods used in many studies. Here, indicate whether each material, system or method listed is relevant to your study. If you are not sure if a list item applies to your research, read the appropriate section before selecting a response.

Materials & experimental systems

n/a	Involved in the study	
☐	☒	Antibodies
☒	☐	Eukaryotic cell lines
☒	☐	Palaeontology and archaeology
☒	☐	Animals and other organisms
☐	☒	Human research participants
☒	☐	Clinical data
☒	☐	Dual use research of concern

Methods

n/a	Involved in the study	
☒	☐	ChIP-seq
☒	☐	Flow cytometry
☒	☐	MRI-based neuroimaging

Antibodies

Antibodies used	Antigen/Target, Host, Catalog Number<br>KRT18, Mouse Abcam, ab668<br>SFTPC, Mouse ThermoFisher, PA5-71680<br>EPCAM, Mouse ThermoFischer, MA5-12436<br>SCGB1A1, Mouse Santa Cruz Biotechnology, sc-365992<br>ELF5, Rabbit ThermoFisher, 720380<br>TMPRSS2, Mouse Sigma, HPA035787-100UL<br>ACE2, Rabbit Abcam, ab15348<br>AlexaFluor, 488 Goat anti-Rabbit IgG (H+L) Goat Life Technologies, A11034<br>AlexaFluor, 568 Goat anti-Mouse IgG (H+L) Goat Life Technologies, A11004<br>Dilutions for each antibody can be found in Supplemental Table 8.
Validation	KRT18 - IHP-P, and Flow Cyt by the manufacturer; DOIs: 10.1038/s41556-020-00619-0<br>SFTPC - IF, and WB by the manufacturer; DOIs: 10.3892/ijmm.2020.4791<br>EPCAM - ICC/IF, IHC, Flow Cyt by the manufacturer; DOIs: 10.1186/s13058-017-0843-4 and 10.1016/j.stemcr.2016.12.023<br>SCGB1A1 - WB, and IHC-P by the manufacturer; DOIs: 10.1172/JCI137750 and 10.1038/s41467-021-26770-2<br>ELF5 - ICC/IF, WB, ChIP, and ChIP-qPCR by the manufacturer<br>TMPRSS2 - IHC-P, WB, RNA-seq, and Recombinant Expression by the manufacturer; DOIs: 10.1016/j.cell.2020.02.052 and 10.15252/embj.2020106267

ACE2 - WB, IF, and IHC-P by the manufacturer; DOIs: 10.1084/jem.20202135 and 10.1038/s41467-021-21213-4
AF 488 - Affinity Purified, Cross-Adsorbed, ICC/IF by manufacturer; DOIs: 10.3389/fncel.2019.00001
AF 568 - Affinity Purified, Cross-Adsorbed, ICC/IF by manufacturer; DOIs: 10.1038/s41467-019-09437-x

Human research participants

Policy information about studies involving human research participants

Population characteristics	Tissue samples of the olfactory mucosa and lung from control and COVID-19 patients were collected from the BrainBank/ Biobank of the Department of Neuropathology at the Charité – Universitätsmedizin Berlin (patients) or purchased from OriGene (TissueFocus) and Tissue Solutions (controls). Donor characteristics are listed in Supplementary Table 7. Briefly, 5 men and 3 women died at the age between 31 and 81.
Recruitment	Deceased COVID-19 patients were collected by the BrainBank/Biobank of the Department of Neuropathology at the Charité – Universitätsmedizin Berlin. Patient samples were tested with a Spindiag Rhonda PCR rapid COVID-19 test. Patients with COVID-19 were further stratified as either "rapid" or "later death" based on their disease duration (rapid ≤ 14 days, later death > 14 days). The small sample size limits generalization of observations, but is unlikely to affect results about ELF5-tissue distribution.
Ethics oversight	This study was approved by the local ethics committees (EA1/144/13, EA2/066/20 and EA1/075/19) as well as by the Charité–BIH COVID-19 research board and is in compliance with the Declaration of Helsinki; autopsies were performed on the legal basis of §1 of the Autopsy Act of the state Berlin and §25(4) of the German Infection Protection.

Note that full information on the approval of the study protocol must also be provided in the manuscript.
